# Functional heterogeneity of CD4^+^ T cells in liver inflammation

**DOI:** 10.1007/s00281-021-00881-w

**Published:** 2021-08-31

**Authors:** Franziska Muscate, Anna Woestemeier, Nicola Gagliani

**Affiliations:** 1 Department of General, Visceral and Thoracic Surgery, University Medical Center Hamburg-Eppendorf, Hamburg, Germany; 2 Department of Medicine, University Medical Center Hamburg-Eppendorf, Hamburg, Germany; 3 Hamburg Center for Translational Immunology (HCTI), University Medical Center Hamburg-Eppendorf, Hamburg, Germany

## Abstract

CD4^+^ T cells play an essential role in orchestrating adequate immunity, but their overactivity has been associated with the development of immune-mediated inflammatory diseases, including liver inflammatory diseases. These cells can be subclassified according to their maturation stage, cytokine profile, and pro or anti-inflammatory functions, i.e., functional heterogeneity. In this review, we summarize what has been discovered so far regarding the role of the different CD4^+^ T cell polarization states in the progression of two prominent and still different liver inflammatory diseases: non-alcoholic steatohepatitis (NASH) and autoimmune hepatitis (AIH). Finally, the potential of CD4^+^ T cells as a therapeutic target in both NASH and AIH is discussed.

## Introduction

In healthy conditions, the liver can tolerate the influx of food- and bacterial-derived antigens and pathogen-associated molecular patters (PAMPs). This is possible due to several immunoregulatory mechanisms including a tight control of T cell activation by, for example, regulatory T cells [14, 68, 90]. However, a variety of environmental and genetic factors such as viral infection, alcohol, obesity, and HLA risk alleles can favor inflammatory liver diseases of which non-alcoholic steatohepatitis (NASH) and autoimmune hepatitis (AIH) are among the most common ones creating a severe public health challenge [2, 86, 109, 113, 138].

Obesity and metabolic syndrome promote accumulation of lipids in the liver and thereby cause NAFLD (non-alcoholic fatty liver disease). The accumulation of lipids is accompanied by cellular stress and leads, in some patients, to tissue damage and inflammation (NASH, non-alcoholic steatohepatitis) [49]. NASH development has been associated with high intake of nutrients, but also with an altered microbiota [11, 131]. A potentially detrimental effect of the intestinal microbiota on the progression from NAFLD to NASH has so far only been shown in mouse models. While germ-free mice on a high-fat diet (HFD) are protected from NASH, transplantation of stool from dysbiotic mice accelerates disease [4, 42, 121]. Translocation of bacterial antigens due to increased gut leakiness has also been suggested to link the intestine and the liver and thereby to further enhance inflammation and disease progression [30, 99].

AIH is characterized by destruction of the hepatic parenchyma by an autoreactive immune response. Clinical manifestations of early AIH are rather heterogeneous across patients, but characteristic to all of them is a progressive and detrimental disease with high titers of auto-antibodies and liver infiltrating plasma cells [5, 71, 82]. Tissue damage in AIH is directly mediated by immune cells and is usually accompanied by stronger infiltration of lymphocytes compared to NASH patients [125].

Both NASH and AIH can be followed by cirrhosis and hepatocellular carcinoma (HCC) [1, 122]. HCC caused roughly 782 000 deaths worldwide in 2018 [13].

The mechanism driving both NASH and AIH is not clear, but there is evidence of an important role of T cells.

T cells are the central orchestrators of inflammatory responses. Indeed, in an experimental mouse model of NASH, the blockade of CD4^+^ T cell infiltration into liver and small intestine protects the mice from the development of NASH [100]. In this model, an increased number of peripheral T cells express the integrins α4β5 when comparing MCD diet fed mice to those on a normal diet. At the same time, the expression of the α4β5 ligand MAdCAM-1 is elevated in the gut and liver tissue. The expression of MAdCAM-1 is dependent on the microbiota since antibiotic treatment reduces its expression. Infiltration of CD4^+^ T cells in both tissues can be blocked by α4β5 antibodies and protect from liver inflammation [100].

Evidence of the role of CD4^+^ T cells in AIH were provided using different mouse models. Conditional expression of autoantigen in the liver was shown to cause spontaneous development of AIH by autoreactive CD4^+^ T cells. Similarly, a defect in central tolerance due to a deletion of medullary thymic epithelial cells or by thymectomy of neonatal PD1^−/−^ mice caused AIH in a T cell-dependent mechanism. Finally, transfer of CD4^+^ T cells of mice suffering from AIH could induce liver inflammation in recipient mice.

Considering that T cells, in particular CD4^+^ T cells, play a key role in both NASH and AIH, here we will dissect the contribution of the different subsets of CD4^+^ T cells in the pathogenesis of these immune-mediated inflammatory liver diseases.

## Naïve CD4^+^ T cells

Priming of naïve CD4^+^ T cells usually occurs in secondary lymphoid organs such as spleen and lymph nodes. Naïve CD4^+^ T cells are found in circulation, and by expressing a particular combination of receptors (e.g., CCR7 and CD62L), they are able to home to the lymphnodes (e.g., CCR7) but not to enter tissue [18, 23, 47, 66, 102]. However, in contrast to other tissues, the architecture of the liver allows interaction of blood circulating T cells with antigen-presenting cells of the liver in the sinusoids [8, 22, 129]. Consequently, the liver might not only represent an additional site of T cell priming, but its unique environment might also predetermine the fate of CD4^+^ T effector cells during inflammatory liver disease. The anatomical site of priming of naïve CD4^+^ T cells is proposed as an important factor determining subsequent CD4^+^ T cell polarization and their capacity to infiltrate tissues. Recently, we have demonstrated the presence of resident naïve like T cells in human livers; however whether these cells are primed in the tissue and whether this determines their potential pathogenic fate remains still unclear, especially in the context of liver inflammation. Despite the lack of data on the above mentioned concept, there is data on the possibility that naïve T cells can be directly primed in the liver.

Professional antigen-presenting cells in the liver are liver sinusoidal endothelial cells (LSEC) and Kupffer cells, both lining the liver sinusoids which makes them easily accessible for blood circulating naïve T cells. In in vitro culture, LSEC are able to efficiently present antigens and activate naïve CD4^+^ T cells. In this system, CD4^+^ T cells start producing the cytokines IL-10, IL-4, and IFN-γ [60]. However, CD45^−^ CD31^bright^ cells, which might either represent LSEC or vascular endothelial cells, were not able to activate naïve CD4^+^ T cells [58]. Therefore, further studies are needed to fully elucidate the role of LSEC in priming naïve CD4^+^ T cells in vivo.

Kupffer cells are macrophages specialized to the liver environment. They reside in the sinusoids and express high levels of MHCII and co-stimulatory molecules and are able to activate naïve CD4^+^ T cells even though to a lesser extent than splenic dendritic cells [75, 135].

Transgenic mouse models expressing specific antigens in the liver have been used to study T cell activation in vivo. Using a mouse model of antigen (i.e., ovalbumin) specific activation, the activation of antigen-specific CD8^+^ T cells was observed, while the activation of CD4^+^ T cells failed [25, 130]. However, another study suggests that the abovementioned effect is at least partially dependent on the type of antigen, since in a similar transgenic mouse model in which antigen derived from mycobacterium instead of ovalbumin is expressed, naïve CD4^+^ T cells could be activated in the liver by Kupffer cells [118].

Additionally, ectopic expression of neural antigen in the liver leads to development of naive CD4^+^ T cells into Foxp3^+^ T_REG_ cells, which in turn protect from experimental autoimmune encephalomyelitis (EAE)/immunopathology in the central nervous system (CNS) [19, 78]. This suggests that naïve CD4^+^ T cells can infiltrate the liver and, at least under physiological conditions, will acquire a regulatory phenotype promoting tolerance to antigen present in the liver.

Less accessible than LSECs and Kupffer cells are hepatic stellate cells (HSC) which are located in the perisinusoidal space. Despite being less accessible, in vitro cultures and adoptive transfer experiments in which mice are lacking MHCI showed that HSC cells can present antigen to T cells [128].

**Fig. 1:**
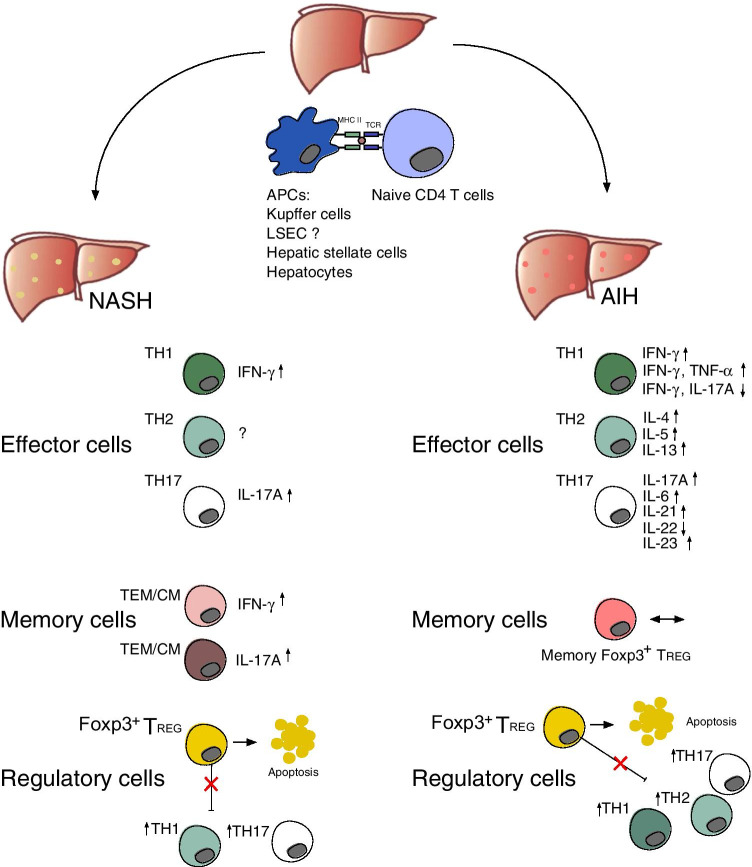
Liver CD4^+^ T cells and their role in NASH and AIH. Naïve CD4^+^ T cells can be primed directly in the liver by different types of antigen-presenting cells (APC) and then mature into effector T cells with different polarization states, namely T_H_1, T_H_2, and T_H_17. The cytokine profile of the effector cells has been associated with the development of NASH and AIH. Effector T cells can form effector memory (T_EM_) and central memory T (T_CM_) cells, and their cytokine profile has also been associated with the development of NASH. In AIH, the frequency of memory T_REG_ cells was found to be not significantly different between AIH patients and healthy subjects. Finally, it has been proposed that Foxp3^+^ T_REG_ cells can undergo apoptosis in the inflamed liver and thus, probably, unleash the pathogenic activity of the effector T cells

During inflammatory conditions, the portfolio of antigen-presenting cells in the liver might be expanded to hepatocytes. Hepatocytes express no or low levels of MHCII during physiological conditions. However, MHCII expression on hepatocytes can be detected in alcoholic and non-alcoholic hepatitis [77]. In a transgenic mouse model expressing MHCII on hepatocytes, CD4^+^ T cells can indeed be activated [45] (Fig. 1). Whether priming of naïve T cells by hepatocytes is taking place in NASH or AIH still needs to be confirmed.

Additionally, the impact of naïve T cells activated by hepatocytes during chronic inflammation on the disease progression needs further investigation. A study performed in a transgenic mouse model expressing antigen on hepatocytes observed indeed an impaired T cell response during LCMV infection. In this model, T cells showed a decreased INF-γ production and transgenic mice were impaired in virus control [127].

In summary, data from in vitro cultures and transgenic mouse models suggest that naïve CD4^+^ T cell can be primed in the liver. However, the consequences of this ectopic activation for human inflammatory liver diseases need to be further investigated.

## Effector T cells

It has been suggested that CD4^+^ effector T cells play important roles in both protecting the liver from infections and also causing hepatocellular damage and autoimmunity [97]. Early stages of liver inflammation are dominated by CD4^+^ effector T cells and followed by a cytotoxic CD8^+^ T cell response [91, 112, 116]. Effector CD4^+^ T cells can acquire different cell states (i.e., T_H_1, T_H_2, and T_H_17 cells), here referred to as subsets, which are characterized by different cytokine profiles. Moreover, it has been shown that CD4^+^ T cell subsets can display a mixed phenotype characterized by the concomitant features of different polarization states, e.g., cytokines, and even potentially loose their originally polarization state acquiring a different one. For example, T_H_17 cells can acquire both a T_H_1 phenotype under chronic inflammation [39, 46, 57] and an anti-inflammatory phenotype during the resolution of the inflammation [34, 132]. The above described cellular phenomenon is here defined as T cell plasticity.

The different role of CD4^+^ T cell subsets in NASH and AIH patients has begun to be elucidated (Fig. 1). However, the role of plasticity in the context of AIH and NASH has scarcely been investigated.

Below we provide a summary of the role of the different CD4^+^ T cells subsets in NASH and AIH.

### T_H_1 cells

The infiltration of the liver by T_H_1 cells, which are characterized by the production of IFN-γ, was shown to correlate also strongly with disease progression and liver injury of AIH patients [108].

T_H_1 cells were found to be enriched in the liver of NASH patients [7, 50]. Furthermore, investigating the NASH hepatic gene signature, IFN-γ response pathway genes showed the highest enrichment [38]. In the peripheral blood of NAFLD patients, Rau et al. also showed an increase in T_H_1 cells compared to healthy controls [101]. The potential pathogenic role of IFN-γ in the liver is probably attributed to its multiple detrimental functions, including induction of hepatocyte apoptosis and cell cycle arrest [111], induction of expression of chemokines such as CCR2 and their receptors on liver cells [51], and activation of Kupffer cells [142].

CD4^+^ T cells can adapt in response to a changing environment and therefore exhibit different polarization states [34, 132]. However, studies exploring the role of T cells in NASH are so far limited to selected key cytokines, such as IFN-γ, and do not investigate the potential plasticity of T cells.

In the liver of AIH patients, an increase of IFN-γ–producing cells was observed [74, 139, 140]. In addition, in a concanavalin A (ConA) mouse model of immune-mediated liver injury, a reduction of serum IFN-γ levels lead to decreased liver injury [141].

Furthermore, the presence of T_H_17 cells and T cells co-producing IFN-γ and TNF-α was reported in AIH [12, 103]: Findings indicated that TNF-α-producing CD4^+^ T cells were significantly expanded, both in blood and liver of AIH patients. However, the majority of the TNF-α-producing CD4^+^ T cells in AIH also co-produced IFN-γ, suggesting that these cells might represent a pathogenic activation state of T_H_1 cells [12].

CD4^+^ T cells co-producing IFN-γ and IL-17A (T_H_1/T_H_17 cells) were found to be decreased in the early stages of AIH pathogenesis in the blood, consistent with a working hypothesis of an enhanced recruitment of cells into the liver. Interestingly, AIH patients under standard immunosuppression (corticosteroids, azathioprine) failed to correct these T_H_1/T_H_17 imbalances in the blood and a persistent infiltration in the liver was observed, demonstrating that a deeper immunological restoration of tolerance does not occur despite satisfactory resolution of hepatitis [103].

These findings indicate that AIH is not only associated with classical T_H_ cell subsets, but rather with a larger spectrum of mixed T_H_ cell subsets with different polarization states, which should be further investigated (Fig. 1).

### T_H_2 cells

T_H_2 cells ensure protective immunity against helminthic infections and play a key role in the pathogenesis of allergic diseases [124]. In the liver, T_H_2 cells were shown to have a strong pro-fibrogenic effect, and inhibition of IL-13 signaling blocks fibrosis development [95]. Few studies have thoroughly investigated this subset in the context of NAFLD and AIH and their role in these diseases remains unknown.

Rau et al. described an increase in circulating T_H_2 cells of NAFLD patients compared to healthy normal-weight controls, who were not matched for age [101]. Interestingly, 12 months after bariatric surgery, the T_H_2/ Foxp3^+^ T_REG_ ratio was decreased. However, other authors did not find any differences in T_H_2 numbers, neither in peripheral blood nor in the liver when they compared NASH patients and NAFLD patients or controls [31, 50]. To our knowledge, the involvement of the T_H_2 subset has not been thoroughly investigated in an animal model of NAFLD.

In AIH, the role of T_H_2 cells still remains elusive. Early studies showed that the T_H_2 cytokines IL-5 and IL-13 were present in the late cirrhotic stage of AIH patients [24, 26, 91, 92]. In type I autoimmune hepatitis in children, an increased mRNA expression of *Il4* was observed in liver samples [21]. Furthermore, it is known that the cytokines IL-4 and IL-6 can regulate B-cell activation and promote the production of antinuclear antibodies (ANA) and anti-smooth muscle antibodies (SMA) [110]. However, no significant differences in *Il4* mRNA expression levels between patients and healthy subjects were observed in peripheral blood mononuclear cells (PBMCs) [6].

In mouse models, IL-4 producing T_H_2 cells play an essential role in inducing ConA-immune-mediated liver injury via activation of STAT6. STAT6 upregulates the expression of the chemoattractant eotaxin in hepatocytes and sinusoidal endothelial cells and induces IL-5 expression, resulting in eosinophil and neutrophil recruitment into the liver and leading to hepatitis [52] (Fig. 1).

### T_H_17 cells

T_H_17 cells are characterized by the production of IL-17A, IL-17F, and IL-22 and are believed to play an important role in the development of a variety of autoimmune diseases [64].

T_H_17 cells were shown to be present in larger numbers in the liver of NASH patients in comparison to healthy controls [36, 101, 123]. Moreover, Rau et al. report a decrease of T_H_17 cells in peripheral blood, as well as in the T_H_17/ Foxp3^+^ T_REG_ ratio, when NASH patients were re-evaluated 12 months after bariatric surgery [101].

Furthermore, T_H_17 cells were also shown to be present in larger numbers in the liver and peripheral blood of NAFLD mouse models [81, 106, 115]. The IL-17A and IL-17F axis was shown to be important in the development and progression of NASH. IL-17RA^−/−^, IL-17A^−/−^, and IL-17F^−/−^ mice exhibited decreased steatohepatitis and hepatocellular damage [35, 41, 106]. In line with these findings, the use of an anti-IL-17 monoclonal antibody significantly improved liver function, attenuated hepatic lipid accumulation, suppressed Kupffer cell activation, and decreased pro-inflammatory cytokine levels in a model of HFD induced NAFLD [133, 134]. Simultaneous blocking of CD25 or IL-17A and IL-17F shifted the T_H_17/Foxp3^+^ T_REG_ imbalance from MCD diet-induced T_H_17 dominance to Foxp3^+^ T_REG_ dominance and also decreased hepatic steatosis and inflammation [73].

Moreover, multiple experimental murine and in vitro models showed that IL-17A administration can lead to an increase in hepatic steatosis [41, 44]. IL-17A was reported to have a pro-fibrotic effect through activation of hepatic stellate cells [114] and an in vitro study showed that IL-17A enhances the expression of pro-fibrotic genes (e.g., ACTA2 and COL1A1) through an upregulation of TGF-β receptor [28].

AIH has been associated with IL-17A expression [12, 37, 63, 70, 139, 140], although the role of T_H_17 cells in the pathogenesis of AIH remains controversial.

The frequency of circulating T_H_17 cells and the expression of the key transcription factor for these cells, RORɣt, were elevated in PBMCs of AIH patients [6, 136, 139, 140]. In the liver of AIH patients, the frequency of IL-17A producing cells and the expression of T_H_17-related cytokines (IL-23, IL-21, IL-1β, and IL-6) was also significantly elevated [139, 140]. Interestingly, the duration and severity of hepatitis may be dependent on T_H_17 cells in AIH [117].

In ConA-induced liver injury, IL-17A-deficient mice develop the same level of liver injury as wild-type mice [137]. In contrast, the results from two independent research studies indicated that IL-17A-deficient mice had a significant reduction in liver injury compared with wild-type mice [63, 88]. The reason for the discrepancy between the findings is not clear, but the authors speculate that they could be attributed to the different environment of the animal facilities that may affect IL-17A^−/−^ mice.

While IL-17A and IL-17F seem to play an important role in inducing liver inflammation via stimulating multiple types of liver non-parenchymal cells to produce pro-inflammatory cytokines and chemokines, IL-22 appears to be an important factor in promoting hepatocyte survival and proliferation. It was demonstrated that treatment with IL-22 prevents, while treatment with IL-22 neutralizing antibodies enhances ConA-induced liver injury [98]. The hepatoprotective role of IL-22 in T-cell hepatitis was also confirmed by other studies using IL-22-deficient mice [59, 137] (Fig. 1).

## Memory T cells

Following the expansion phase of effector T cells, three main populations of memory cells can be recognized: central memory T cells (T_CM_), effector memory T cells (T_EM_), and tissue-resident memory T cells (T_RM_). At present, these memory T cell subsets are primarily characterized by their phenotype, migratory properties, and tissue homing patterns, which in many instances imply unique functional attributes [3, 87]. Memory T cells are not only involved in promoting physiological immunity, but also in promoting autoimmune responses. Due to rapid pro-inflammatory qualities of T_RM_ cells, they can lead a misguided action and result in immunopathology [62]. The role of CD4^+^ memory T cells in NASH and AIH has scarcely been investigated and the phenotypic markers used to define memory T cells are not consistent between studies.

In NASH, patients showed increased numbers of IFN-γ^+^ memory (CD45RO^+^) CD4^+^ and CD8^+^ T cells compared with controls, while numbers of CD4^+^ and CD8^+^ CD45RA^+^ subsets were decreased [50]. One of the molecular mechanisms driving T cell infiltration into the liver is increased chemotaxis, as peripheral CD4^+^ T cells from obese mice and NASH patients migrate more readily toward the chemokine CXCL12 compared to T cells from healthy mice or healthy donors [10]. In line with this finding, a longitudinal analysis of peripheral blood of humanized mice showed that central memory (CCR7^+^CD45RA^–^) and effector memory (CCR7^–^CD45RA^–^) CD4^+^ T cells and their associated cytokines IL-17A and IFN-γ expanded with time and infiltrated the liver (Her et al.,2020).

## Regulatory T cells

Regulatory T cells and the capacity of some effector cells to convert into regulatory T cells provide, among others, key mechanisms to establish peripheral tolerance. CD4^+^ T cells with regulatory function can be divided into at least two subsets: Foxp3^+^ T_REG_ cells and Foxp3^−^ IL10^+^ type 1 regulatory T (T_R_1) cells. Both cell subsets have been described in the context of liver tolerance.

In an hepatitis B virus (HBV) carrier mouse model, Kupffer cells induce T_R_1 cells rather than Foxp3^+^ T_REG_ cells [133, 134]. Transfer of CD4^+^ T cells from HBV carrier mice confers systemic tolerance to HBV antigen in recipient mice. This tolerogenic effect is dependent on IL-10 expression. However, the majority of reports on regulatory T cell subsets in the liver focuses on Foxp3^+^ T_REG_ cells. Specifically in the context of NASH and AIH, the role of T_R_1 cells has not yet been investigated.

A first indication for the importance of Foxp3^+^ T_REG_ cells in liver tolerance is provided by the observation that injection of anti-CD25 antibodies leads to rejection of liver transplants in mice [67]. Foxp3^+^ T_REG_ cells can be induced both by hepatocytes and HSC in in vitro cultures. The induction is favored by the presence of TGF-β and Notch signaling by hepatocytes and IL-2 and retinoid acid receptor in the context of HSC co-culture [17, 27, 54]. In vivo, expression of antigen delivered by AAV vectors in hepatocytes leads to tolerance towards the antigen which is presumably mediated by Foxp3^+^ T_REG_ and Kupffer cells [14]. The tolerogenic function of the liver can be utilized for therapeutic purposes in mouse models. Indeed, application of nanoparticles delivering neuronal peptide to LSEC protects from immunopathology in EAE through the conversion of T cells to Foxp3^+^ T_REG_ in a TGF-β-dependent mechanism [19]. Hence, Foxp3^+^ T_REG_ cells are generated in the tolerogenic liver environment and can support tolerance in the CNS.

During the inflammatory condition of NASH, Foxp3^+^ T_REG_ cells play a key role in disease control. Depletion of Foxp3^+^ T_REG_ cells in a mouse model of NASH aggravates disease [105]. The inflammatory environment during NASH impairs Foxp3^+^ T_REG_ cell survival. Oxidative stress, TNF-α, and type I interferon produced by Kupffer cells and dendritic cells during NASH promote apoptosis of Foxp3^+^ T_REG_ cells [80, 105]. Oxidative stress seems to preferentially induce apoptosis in Foxp3^+^ T_REG_ cells and consequently shifts the ratio of effector T cells to Foxp3^+^ T_REG_ cells towards effector T cells [80]. More precisely, both the ratio of T_H_17 and T_H_2 effector cells to T_REG_ cells have been associated with severity of inflammation, i.e., the progression of NAFLD to NASH. Bariatric surgery could recover the imbalance of Foxp3^+^ T_REG_ and effector T cells [101]. Overall, Foxp3^+^ T_REG_ cells seem to have a protective role during disease progression in mouse models of NASH. However, the protective function of Foxp3^+^ T_REG_ cells might be limited by their increased apoptosis rate during inflammation.

The role of Foxp3^+^ T_REG_ in AIH disease progression is still discussed.

Clearly, adoptive transfer of Foxp3^+^ T_REG_ cells can overcome inflammation in experimental mouse models of AIH including a model of AIRE-mutation, xenoimmunization with human autoantigen, and ConA-induced liver injury [40, 48, 65]. However, it remains unclear whether Foxp3^+^ T_REG_ cells in AIH patients are impaired in number. In part, conflicting results can be explained by different approaches to define Foxp3^+^ T_REG_ cells. In a study investigating Foxp3^+^ T_REG_ defined by CD25^hi^ expression, the authors observed diminished Foxp3^+^ T_REG_ cells in AIH patients in comparison to healthy controls [76]. Importantly, CD25 is not only expressed by Foxp3^+^ T_REG_ but also by activated effector T cells. Hence, other studies have distinguished Foxp3^+^ T_REG_ and T effector cells in human more precisely by combining CD25, CD127, and Foxp3. Using this more stringent definition of Foxp3^+^ T_REG_ cells, no difference in the frequency of Foxp3^+^ T_REG_ in AIH patients compared to healthy controls was observed [94]. Also, the frequency of memory Foxp3^+^ T_REG_ cells defined as CD25^+^CD127^‐^FOXP3^+^ CD45RA^−^ were not significantly different between AIH patients and healthy subjects [103].

Moreover, the frequency of Foxp3^+^ T_REG_ cells in the blood positively correlates with severity of inflammation within the group of AIH patients [94]. In line with this observation, intrahepatic Foxp3^+^ T_REG_ cells in untreated AIH patients are rather enriched, and the number of these cells decreases during immunosuppression [116].

Another study focused on the Foxp3^+^ T_REG_ to effector T cell ratio rather than the plain number of these cells. The authors found a dysbalance of Foxp3^+^ T_REG_ and effector T cells in AIH patients [69]. One possible explanation comes from data suggesting that Foxp3^+^ T_REG_ cells are more prone to undergo apoptosis in active AIH patients [55]. In addition, Foxp3^+^ T_REG_ cells from a proinflammatory enviroment exhibit lower levels of the anti-apoptotic molecule c-Flip and high expression of CD95, indicating elevated susceptibility to Fas-mediated apoptosis [20, 96]. Additionally, Foxp3^+^ T_REG_ cells require the cytokine IL-2 for survival, and the concentration of IL-2 was shown be lower in diseased liver as compared to healthy liver [20]. Indeed, in vitro studies stimulating PBMCs or liver infiltrating lymphocytes from patients with autoimmune liver diseases with low doses of IL-2 showed improved survival and function of Foxp3^+^ T_REG_ [53]. In line with this, in an experimental mouse model of AIH, treatment with complexed IL-2/anti-IL-2 could increase the number of Foxp3^+^ T_REG_ and diminish disease severity [16] (Fig. 1).

In conclusion, on the one hand, an increased susceptibility of Foxp3^+^ T_REG_ to undergo apoptosis during a pathological liver inflammation might explain the restrict capacity of these cells to expand in equal proportion to effector T cells. On the other hand, the preliminary and promising preclinical and clinical studies testing either Foxp3^+^ T_REG_ cell therapy or complexed IL-2 suggest that Foxp3^+^ T_REG_ are definitely an interesting therapeutic target for AIH and probably also for NASH patients.

## Therapeutic approaches

While homeostatic inflammation is an aspect of an healthy liver, a lack of resolution or chronic liver injury leads to progressive liver fibrosis and permanent liver damage. Eventually a chronic liver inflammation leads to HCC and to the death of the patients.

For the treatment of NASH, there is not a single drug approved by the Food and Drug Administration (FDA) or European Medicines Agency (EMA). Standard AIH treatment consists of immunosuppressive therapy; however more than 70% of patients relapse when treatment withdrawal is attempted, suggesting a persistence of pathogenic cells, such as autoreactive CD4^+^ T cells [43, 117].

Approaching liver disease as a range of overlapping pathways leading to the dysregulation of homeostatic inflammatory processes provides novel avenues for the development of future therapies targeting inflammation and resolution within the liver.

There are only a few therapeutic approaches in NASH targeting T cells. CCR2 was shown to play an important role in T cell differentiation [79]. In hepatic inflammation and fibrosis the dual CCR2/CCR5 chemokine receptor antagonist (Cenicriviroc) has been efficient [29] and is therefore been investigated in current phase III clinical trials in patients with NASH and fibrosis (ClinicalTrials.gov Identifier: NCT03028740). However, a decrease in fibrosis but no NASH resolution was observed in a phase IIb trial (ClinicalTrials.gov Identifier: NCT02217475).

Since TNF-α producing cells, including T cells, were shown to be involved in the pathogenesis of AIH, a study by Weiler-Norman and colleagues reported the first series of AIH patients who were treated with infliximab, an antibody targeting TNF-α. The study included 11 difficult-to-treat AIH patients to whom the standard treatment did not lead to remission. Here, infliximab treatment led to a reduction of inflammation [56, 126].

Of note, a retrospective statistical analysis of different clinical studies all including patients treated with anti-TNF-α, and a single center report of 8 cases, showed that anti-TNF-α treatment associates with liver damage [33, 104]. Therefore, further clinical studies testing the effect of anti-TNF-α treatment are urgently needed in AIH patients.

Anecdotal clinical observations with off-label use of ustekinumab, a pharmacological antagonist of the IL-23/IL-17 axis [32], do not indicate a significant effect on AIH activity.

The association between regulatory T cell deficiency and inadequate immune tolerance in AIH sparked rationale to treat autoimmune diseases by the administering autologous Foxp3^+^ T_REG_ cells. Foxp3^+^ T_REG_ cell directed therapy, though ex vivo expansion or IL-2 administration, is increasingly tested in the context of posttransplant tolerance [15, 107, 119, 120] and type 1 diabetes mellitus [9, 84]. Here, the safety and feasibility of Foxp3^+^ T_REG_ cell therapy in humans was shown, and the evidence suggested potential improvements in clinical, biochemical, and immunological status with Foxp3^+^ T_REG_ cell therapy. In AIH patients, it has been shown that autologous Foxp3^+^ T_REG_ cell therapy is feasible and safe, and interestingly a strong preferential homing of Foxp3^+^ T_REG_ cells to the liver and spleen was observed for up to 72 h. However, this study was neither designed nor had the statistical power to demonstrate an effect on AIH activity [93]. Also, administration of IL-2 to AIH patients was shown to increase the pool of circulating Foxp3^+^ T_REG_ cells, and it was proven to be safe in the two treated patients [72]. In short, despite these preliminary encouraging data, larger clinical trials targeting Foxp3^+^ T_REG_ cells in AIH patients are urgently needed.

Finally, CXCL9 and CXCL10 were shown to regulate the differentiation of naïve T cells to T_H_1 cells and lead to the migration of immune cells to inflammatory sites [61, 85]. Plasma levels of CXCL9 and CXCL10 increase with advancing disease stage in AIH [89], although this can be reduced with administration of ursodeoxycholic acid (UDCA) in some patients [83]. A multicenter phase-II clinical trial of a humanized anti-CXCL10 antibody in the treatment of primary biliary cholangitis is currently underway. If this shows promise, AIH would also be a potential indication for future therapeutic study using this agent.

In conclusion, there are only few therapeutic approaches targeting T cells in NASH. However, due to the strong T cell activation within this disease, T cells could be a promising target for future therapies. Furthermore, in AIH patients, cell-based therapies, such as regulatory T cell therapy, could finally replace long-term immunosuppression treatments which are still characterized by serious side effects.
